# Transcriptional reprogramming in yeast using dCas9 and combinatorial gRNA strategies

**DOI:** 10.1186/s12934-017-0664-2

**Published:** 2017-03-15

**Authors:** Emil D. Jensen, Raphael Ferreira, Tadas Jakočiūnas, Dushica Arsovska, Jie Zhang, Ling Ding, Justin D. Smith, Florian David, Jens Nielsen, Michael K. Jensen, Jay D. Keasling

**Affiliations:** 1 The Novo Nordisk Foundation Center for Biosustainability, Technical University of Denmark, 2800 Kgs Lyngby, Denmark; 2 Department of Biology and Biological Engineering, Novo Nordisk Foundation Center for Biosustainability, Chalmers University of Technology, 412 96 Gothenburg, Sweden; 3 Department of Genetics, Stanford University School of Medicine, Stanford, CA 94305 USA; 4 Stanford Genome Technology Center, Palo Alto, CA 94304 USA; 5 Joint BioEnergy Institute, Emeryville, CA USA; 6 Biological Systems and Engineering Division, Lawrence Berkeley National Laboratory, Berkeley, CA USA; 7 Department of Chemical and Biomolecular Engineering & Department of Bioengineering, University of California, Berkeley, CA USA

**Keywords:** dCas9, gRNA, VPR, Mxi1, scRNA, Yeast, Isoprenoids, Triacylglycerols, Transcriptional regulation

## Abstract

**Background:**

Transcriptional reprogramming is a fundamental process of living cells in order to adapt to environmental and endogenous cues. In order to allow flexible and timely control over gene expression without the interference of native gene expression machinery, a large number of studies have focused on developing synthetic biology tools for orthogonal control of transcription. Most recently, the nuclease-deficient Cas9 (dCas9) has emerged as a flexible tool for controlling activation and repression of target genes, by the simple RNA-guided positioning of dCas9 in the vicinity of the target gene transcription start site.

**Results:**

In this study we compared two different systems of dCas9-mediated transcriptional reprogramming, and applied them to genes controlling two biosynthetic pathways for biobased production of isoprenoids and triacylglycerols (TAGs) in baker’s yeast *Saccharomyces cerevisiae.* By testing 101 guide-RNA (gRNA) structures on a total of 14 different yeast promoters, we identified the best-performing combinations based on reporter assays. Though a larger number of gRNA-promoter combinations do not perturb gene expression, some gRNAs support expression perturbations up to ~threefold. The best-performing gRNAs were used for single and multiplex reprogramming strategies for redirecting flux related to isoprenoid production and optimization of TAG profiles. From these studies, we identified both constitutive and inducible multiplex reprogramming strategies enabling significant changes in isoprenoid production and increases in TAG.

**Conclusion:**

Taken together, we show similar performance for a constitutive and an inducible dCas9 approach, and identify multiplex gRNA designs that can significantly perturb isoprenoid production and TAG profiles in yeast without editing the genomic context of the target genes. We also identify a large number of gRNA positions in 14 native yeast target pomoters that do not affect expression, suggesting the need for further optimization of gRNA design tools and dCas9 engineering.

**Electronic supplementary material:**

The online version of this article (doi:10.1186/s12934-017-0664-2) contains supplementary material, which is available to authorized users.

## Background

Control of gene expression largely impacts how living organisms adapt to environmental changes, differences in metabolic fluxes, and developmental cell states [1–3]. In order to provide an optimal response to such external and internal cues, eukaryotes orchestrate complex transcriptional programs in multiple genomic loci simultaneously [4].

For development of cell factories, balanced expression between genes encoding native enzymes of metabolic pathways and heterologous genes encoding multi-step biosynthetic pathways have been explored in order to increase productivity [5–7]. Yet, due to the lack of orthogonal and tuneable transcriptional control mechanisms, metabolic engineers often adopt a few somewhat characterized promoters for driving the expression of biosynthetic pathway genes, albeit without predictive understanding of expression levels of single genes crucial for optimal production.

In the budding yeast *Saccharomyces cerevisiae* the mevalonate (MVA) pathway generates precursors isopentenyl diphosphate (IPP) and dimethylallyl pyrophosphate (DMAPP) from acetyl-CoA through seven enzymatic reactions [8]. Several studies have reported the overexpression and downregulation of key MVA pathway genes, including the ones encoding farnesyl pyrophosphate (FPP) synthase (*ERG20*), squalene synthase (*ERG9*), and the HMG-CoA reductase (*HMG1*), in order to increase production of value-added isoprenoids from simple sugars, while simultaneously maintaining ergosterol levels to support growth [9–11].

In another example, triacylglycerols (TAGs) are key molecules for cell functioning as essential energy storage compounds, and also potential industrial feedstocks for the production of food ingredients, oleochemicals and biodiesel [12, 13]. TAG biosynthesis involves several genes from the lipid metabolic pathway, including those encoding the delta-9 desaturase (*OLE1*) and the diacylglyceride acyl-transferase (*DGA1*) [14, 15], and it has been demonstrated that regulating these two genes dramatically affect lipid composition of the cell [16, 17]. In both examples, increased productivity of isoprenoids and TAGs need to be balanced by cell membrane integrity and growth. This calls for the development and application of new molecular tools to enable testing and identification of optimal expression levels of several genes simultaneously.

In recent years, the use of endonuclease-deficient, yet RNA-binding, Cas9 variants (dCas9) has shown tunable and orthogonal control of gene expression by blocking transcription elongation [18, 19]. The mechanism for directing dCas9 to multiple genes at the same time is identical to that of Cas9, namely by the use of sequence-specific Cas9-binding guide RNAs (gRNAs) [19]. In terms of regulatory action, the repressive nature of dCas9, termed CRISPR (Clustered Regularly Interspaced Short Palindromic Repeats) interference (CRISPRi), has been improved by fusing dCas9 with repressive chromatin modifier domains, like the KRAB (Krüppel associated box) domain of Kox1, and the mammalian transcriptional repressor domain Mxi1 [18]. Likewise, to repurpose dCas9 for gene activation, dCas9 has been coupled to transcription activators, like VP64 and p65AD, thereby upregulating gene expression up to 25-fold when using multiple gRNAs in proximity to the transcription start site (TSS) of single target gene promoters [18, 20–22]. In addition to regulatory action, placing either dCas9 or gRNA expression under the control of an inducible promoter has enabled dose- and time-dependent tuning of target gene expression [19, 23]. More recently, the engineering of gRNA into scaffold RNA (scRNA), which mediates the assembly of dCas9 and other RNA-binding proteins fused to transcription regulatory domains like KRAB and VP64, has enabled both target specificity and regulatory function in the assembled “master-regulator” [24]. Cells expressing such systems allow some genes to be activated and others to be repressed as determined by the scRNAs, and not dCas9 itself [24].

Here we present the use of two dCas9-mediated systems for controlling expression of genes targeted by gRNAs. One system relies on the anhydrotetracycline (aTc)-inducible expression of gRNA expression and dCas9 fused to either Mxi1 or VPR (VP64-p65-Rta) [25] for repression or activation of target gene expression, respectively. The second system has constitutive expression of scRNAs, which link both target site and regulatory action to gene expression. The first system is an extension from the inducible CRISPRi system developed by Smith et al., whereas the constitutive system is further developed from the RNA scaffolding outline developed by Zalatan et al. [23, 24]. We show that the two systems mediate similar quantitative changes in both repression and activation of two target promoters, and that the two systems can be used for single and multiplex transcriptional reprogramming of biosynthetic pathways in yeast. We use budding yeast *S. cerevisiae* as a testbed chassis to test >100 gRNAs positioned along 14 different promoters with basal expression spanning >2.5 orders of magnitude. The gRNAs are able to guide dCas9-mediated activation and repression of gene expression up to 2.5- and 3-fold, respectively. We also demonstrate the impact of single and multiplex gRNA and gRNA strategies for reprogramming expression of multiple genes in the isoprenoid and TAG biosynthetic pathways. Finally, we report targeted multi-gene expression reprogramming to significantly regulate carotenoid production and TAGs profile.

## Results

### Benchmarking two systems for dCas9-mediated gene regulation

**Fig. 1:**
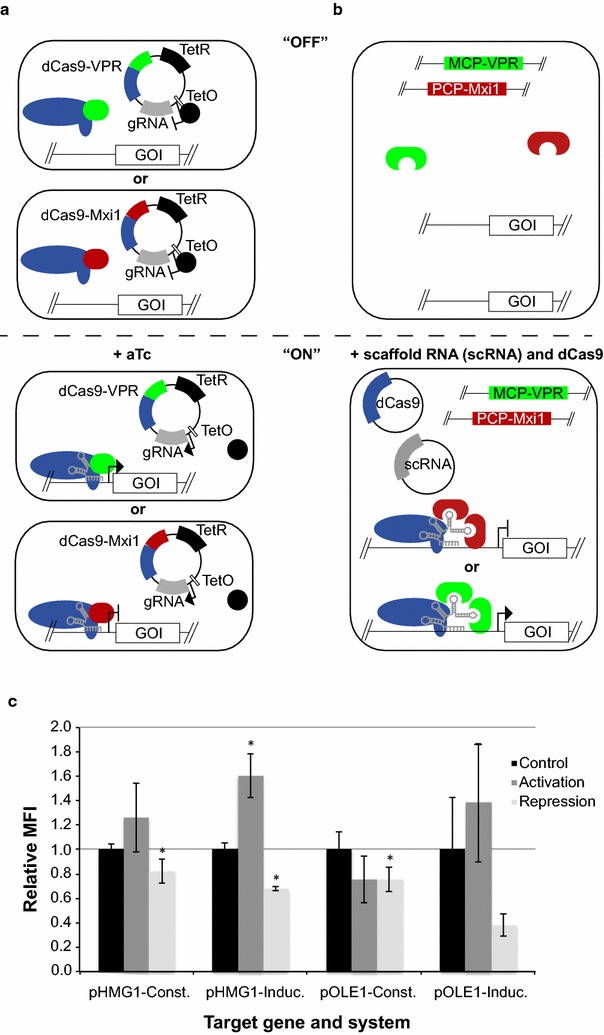
Comparing two dCas9 systems for transcriptional regulation. **a** The anhydro-tetracyclin (aTc) inducible system with gRNA expression controlled by TetO reprograms transcriptional expression with dCas9 directly fused to either VPR or Mxi1 anchoring to promoters of genes of interest (GOI). **b** The constitutive dCas9 and scRNA expression system regulates transcription through orthogonal gRNA scaffold extensions that recruit endogenously transcribed Mxi1 or VPR. Two identical effectors can be recruited per scRNA. Introducing dCas9 and scRNA(s) promote the onset of this system. For both the inducible and the constitutive system Mxi1 (*red*) is used for repression and VPR (*green*) for activation. **c** Benchmarking the inducible and the constitutive system. BioLector data from time-point 24 h are shown for both systems as relative MFI compared to controls. Control levels are shown in *black*, repression in *light grey* and activation in *dark grey*. Results are presented as GFP/OD from targeting the *GFP*-fused promoters HMG1 at position TSS-128 and OLE1 at position TSS-381 in both systems. MFI values are shown as mean ± S.D. from three (n = 3) biological replicate experiments

In order to test dCas9-mediated gene expression in yeast, we initially chose two different approaches (Fig. 1). In one approach, we placed gRNA expression downstream of an aTc-inducible element to control onset of gene regulation. Adding this degree of control allows investigation of immediate effects on gene expression, and impact from timing gene regulation on growing cultures. We used the previously reported construct dCas9-VPR, which cooperatively recruits transcription machinery for activation [25], and dCas9-Mxi1 for repression as previously described [18]. This approach is referred herein as the “inducible system” (Fig. 1a).

In another approach, we leveraged RNA-scaffolds built into the original gRNA structure to facilitate recruitment of either VPR or Mxi1 [24]. VPR and Mxi1 were fused to the orthogonal scaffold-binding domains MCP and PCP, respectively, making simultaneous bi-directional and targeted gene regulation possible (Fig. 1b). In our design, GS-NLS constituted the linker between MCP and VPR, and we used 3× GS as the linker between PCP and Mxi1. Expression of genomically integrated *MCP*–*VPR* and *PCP*-*Mxi1* were controlled by *ADH1* promoters, pADH1. Transcriptional regulation from this system is activated after introducing plasmid-borne dCas9, and a plasmid containing one or more constitutively expressed scRNAs. Herein, we refer to this approach as the “constitutive system”.

We compared the inducible and constitutive systems by targeting two yeast promoters involved in either fatty acid synthesis (pOLE1) or the mevalonate pathway (pHMG1) (Fig. 1c). For our analyses, we designed gRNAs that localize mainly between −200 and +1 nucleotides (nt) relative to the transcription start site (TSS; TSS-200 and TSS+1), which was previously reported to be the region to most likely influence transcriptional regulation using dCas9-mediated reprogramming [23]. For our gRNA designs we assessed self-complementarity and off-targets by CHOPCHOP (http://chopchop.cbu.uib.no) and the algorithm from Smith et al. (http://lp2.github.io/yeast-crispri/) [23, 26] (Additional file 1: Table S4). These software packages also provided predicted nucleosome occupancy and chromatin accessibility. Next, we constructed the pOLE1-*GFP* and pHMG1-*GFP* reporter cassettes and stably integrated these into the yeast genome. Twenty-four hours following either (1) aTc treatment or (2) dilution to OD ~0.2 for the constitutive system, mean fluorescence intensity (MFI) was quantified. The four strains tested using the inducible system were compared to non-induced control strains, while MFI from strains constructed for testing the constitutive system were scored relative to strains with an empty gRNA plasmid. In the aTc-inducible system, expression from pHMG1 was reduced ~1.5-fold and activated ~1.6-fold in strains expressing dCas9-Mxi1 and dCas9-VPR, respectively (Fig. 1c), while the constitutive system conferred ~1.2-fold repression and ~1.3-fold activation using scRNAs for tethering dCas9-Mxi1 or dCas9-VPR, respectively, to the pHMG1 promoter. For pOLE1, the inducible system conferred no significant activation using dCas9-VPR, while induced expression of the gRNA guiding dCas9-Mxi1 resulted in 2.6-fold repression. When targeting pOLE1, the constitutive system did not enable activation, but resulted in significant repression (Fig. 1c).

To evaluate if the dCas9 systems would affect growth when regulating native genes, we tested the growth kinetics of the strains subjected to either of the two regulatory systems. Here, we found no impairment in growth rates relative to control strains upon targeting pHMG1 or pOLE1 for regulation (Additional file 2: Figure S1). On contrary, the inducible system improved growth in some strains. Additionally, when evaluating expression of reporter genes from the two different promoters in the two systems, we observed that changes in expression were maintained for more than 24 h (Additional file 3: Figure S2).

Taken together, using the same gRNAs and dCas9 variant, the relative MFIs were comparable between the two systems for pOLE1. For pHMG1 the inducible system performed slightly better than the constitutive system. Also, for none of the two systems did the synthetic transcriptional reprogramming confer any growth reduction when targeting pHMG1 and pOLE1.

### Regulating gene expression using the constitutive system

**Fig. 2:**
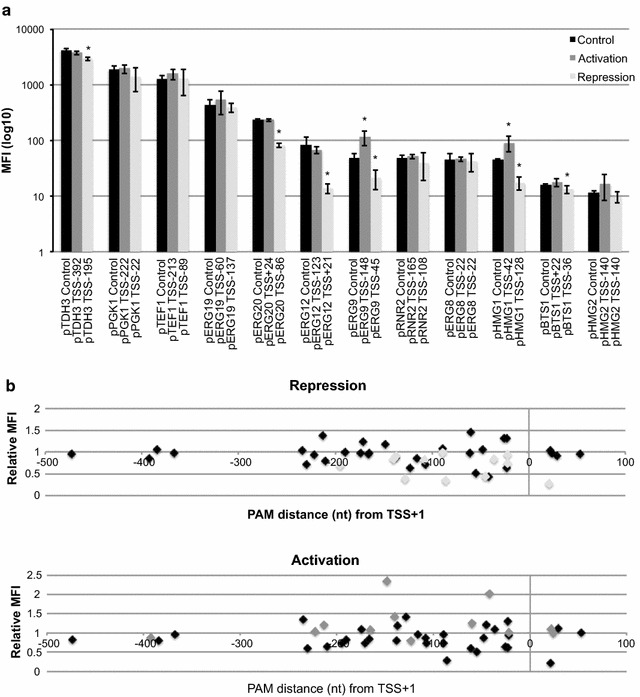
Analyzing transcriptional regulation on yeast promoters using the constitutive system. **a** Log-scaled mean fluorescense intensity for 12 yeast promoters involved in glycolysis and mevalonate metabolism is shown. Activation with MCP-VPR (*dark grey*) and repression with PCP-Mxi1 (*light grey*) is shown next to ‘no gRNA’ controls (*black*; dCas9 expressed) for best performing scRNAs out of 88 for each promoter. *Asterisks* indicate regulation that resulted in significantly altered expression profiles relative to controls (*p < 0.05). MFI values are shown as mean ± S.D. from three (n = 3) biological replicate experiments. **b** Best performing scRNAs for repression (*light grey*) and for activation (*dark grey*) are the same as presented in **a**. Relative MFI (deviation from ‘no gRNA’ control strains Sc-23 to Sc-34 expressing dCas9) conferred by 88 scRNAs (in strains Sc-35 to Sc-122) tethering dCas9 and PCP-Mxi1 or MCP-VPR on a total of 12 promoters is illustrated in *black* relative to PAM distance (nt) from TSS+1

In order to further assess the regulatory potential of the two systems, we tested the constitutive system on a larger set of MVA and glycolytic promoters. The carotenoid pathway from *Xanthophyllomyces dendrorhous* has been extensively used as an efficient screening assay for transcription-based MVA pathway flux in *S. cerevisiae* [27, 28]. Likewise, tuning expression of MVA genes to control flux through the MVA pathway was previously adopted to optimize artemisinin production [29]. Hence, we pursued identifying gRNA entry points for transcriptional regulation of MVA pathway promoters (Fig. 2a). In addition to MVA target genes, our candidate set of promoters also included commonly used strong glycolytic and weak promoters (pTDH3, pTEF1, pPGK1, and pRNR2) [28].

For 12 native yeast promoters we designed 44 gRNAs to combine with the 2× PP7 or 2× MS2 (wt + f6) RNA scaffolds totalling 88 scRNAs (Additional file 4: Figure S3, Additional file 1: Table S4). Twenty-four hours following liquid culture dilution (OD~0.2) of dCas9 and scRNA transformed cells we measured fluorescence intensities from our reporter promoters. From our analyses the regulation capacity was observed to range from threefold repression to 2.3-fold activation for the best-performing scRNAs for each promoter. More specifically, among these designed scRNAs, significant repression of promoter activity of the strong pTDH3 promoter and the medium strength promoters pERG20, pERG12, pERG9 and pHMG1 were observed (Fig. 2a), while significant activation of promoter activity was only observed for pHMG1 and pERG9. However, as evidenced from the total set of tested scRNAs, for some promoters none of the tested scRNAs were able to significantly perturb promoter activity as inferred from reporter assays (Fig. 2a; Additional file 4: Figure S3). In total, 29 out of 88 scRNAs conferred significant regulation (Additional file 4: Figure S3). Our analyses also revealed that even VPR in combination with some scRNAs can confer repression of some promoters (e.g. pERG20 and pERG12), which supports a previous study [30]. Our most impactful scRNA for activation hybridizes to pERG9 (~2.3-fold) at TSS-148 and localizes within a previously identified UAS [31], while the strongest repression on pERG9 (~2.3-fold) was observed from targeting position TSS-66 not overlapping with any annotated promoter elements. Likewise, spanning the TATA-box with the scRNA positioned at TSS-23 did not facilitate activation or repression.

Targeting transcriptional effectors to promoters between TSS-200 and TSS+1 was previously reported to impact expression [23]. Most of our compiled data were sampled in this region, and for the few data points sampled exceeding TSS-200 none conferred significant regulation (Fig. 2b). For scRNAs targeting within the TSS-200 to TSS+1 window, we observed no correlation between the scRNA position and their impact on transcriptional regulation (Fig. 2a, b). In addition to most of the scRNAs targeting sequences upstream of TSS+1, we also selected a few scRNAs targeting downstream TSS+1. For those designed to target dCas9 downstream of the TSS+1, the scRNA targeting the template strand at position TSS+21 of pERG12 was the most impactful resulting in significant repression (Additional file 4: Figure S3). In contrast, we found for several promoters (pERG20, pERG9, pERG8, and pBTS1) in which scRNAs targeted the non-template strand downstream of TSS+1, no significant repression. This finding deviates from previous reports focusing on transcriptional regulation in bacteria [19, 32], but falls in line with similar studies on yeast *ERG11* and *ERG25* transcriptional regulation [23].

To further analyse the observed regulation patterns, we compared the change in promoter activity when using individual scRNA to the predicted nucleosome positioning (Additional file 5: Figure S4) [33]. We found that positioning of scRNAs in regions with predicted low nucleosome occupancy for pHMG1 correlated with higher transcriptional impact in accordance with current literature [23, 34, 35]. For pERG9, designing scRNAs to target nucleosome-free vs. nucleosome-dense regions did not change their impact on transcriptional impact, and investigation of all promoters revealed no overall correlation between transcriptional impact and scRNA positioning in relation to predicted nucleosome positioning (Additional file 5: Figure S4). However, it should be mentioned that for pHMG1 and pERG9, the nucleosome positioning landscape is based on S288C genome data.

In summary, from testing a total of *in silico* designed 88 scRNAs on 12 native yeast promoters using the constitutive system, we observed significant repression of five promoters and activation of two promoters, at a maximum fold-change of 3- and 2.3-fold, respectively. Also, our study identified no clear correlation between transcriptional impact and scRNA positioning in relation TSS, nor correlation between scRNA positioning and predicted nucleosome positioning.

### Regulating expression of TAG biosynthetic genes using the inducible system

From our initial benchmark of the two dCas9 systems the inducible system could efficiently regulate the expression of pOLE1 (Fig. 1c). To further test the applicability of this system, we characterized more gRNAs targeting the pOLE1 promoter. Likewise, we designed several gRNAs targeting the pDGA1. Activation or induction of pOLE1 and pDGA1 activity has previously been shown to increase TAG biosynthesis [16, 17], and we therefore first tested the induction of gRNAs together with expression of dCas9-VPR (Fig. 1a).

**Fig. 3:**
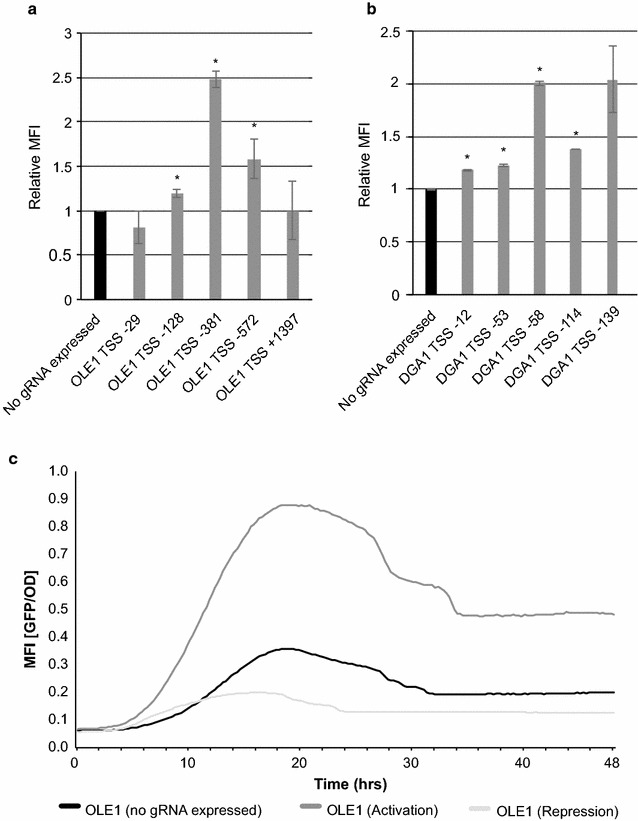
Analyzing transcriptional regulation on yeast promoters using the inducible system. **a** Screening the effect of different gRNAs for pOLE1-*GFP* expression relative to a strain without any gRNA expressed. **b** Screening the effect of different gRNAs for pDGA1-*GFP* expression relative to a strain without any gRNA expressed. Engineered strains were cultivated with 250 ng/mL of aTc. Maximum values for GFP/OD yield were extracted. **c** Mean fluorescence intensities (MFI; GFP/OD) from plasmid-based pOLE1-*GFP* over time targeted with pERA-112 (Sc-170: OLE1 TSS-381 gRNA coupled to dCas9-VPR; *dark grey*) and pERA-117 (Sc-171: OLE1 TSS-381 gRNA coupled to dCas9-Mxi1; *light grey*). All results were obtained from three biological replicates monitored with a BioLector over 48 h. MFI values are shown as mean ± S.D. from three (n = 3) biological replicate experiments. For **a** and **b**, *asterisks* indicate significant regulation relative to controls (*p < 0.05)

Similar to the constitutive system, we coupled pDGA1 and pOLE1 to GFP expression to quantitatively determine interference capacities of several different gRNAs (Fig. 3a). Here, gRNAs targeting pDGA1 at positions TSS-139 and TSS-58 gave the highest upregulation with up to twofold activation (Fig. 3a). For pOLE1, the strongest activation was obtained with a gRNA binding at position TSS-381 (2.5-fold). For pOLE1, the gRNA closest to TSS+1 is positioned at TSS-29, and it slightly down-regulates GFP expression (Fig. 3b). Further analysis of position TSS-381 showed the strongest activation potential around twenty-four hours following aTc-induction (Fig. 3c). As shown in the control, when no gRNA was expressed, a time dependent regulation of pOLE1 was observed (Fig. 3c). This is in line with a time-resolved quantitative analysis performed by Casanovas et al. [36], where it was demonstrated that *OLE1* is highly expressed during early-phase to mid-exponential phase and downregulated from late exponential phase. As such, in addition to endogenous growth phase-dependent regulation, our temporal analysis showed that gRNA-mediated tuning of gene expression is able to downregulate and upregulate pOLE1 activity (Fig. 3c).

### Targeted regulation of biosynthesis by dCas9 and combinatorial gRNA strategies

**Fig. 4:**
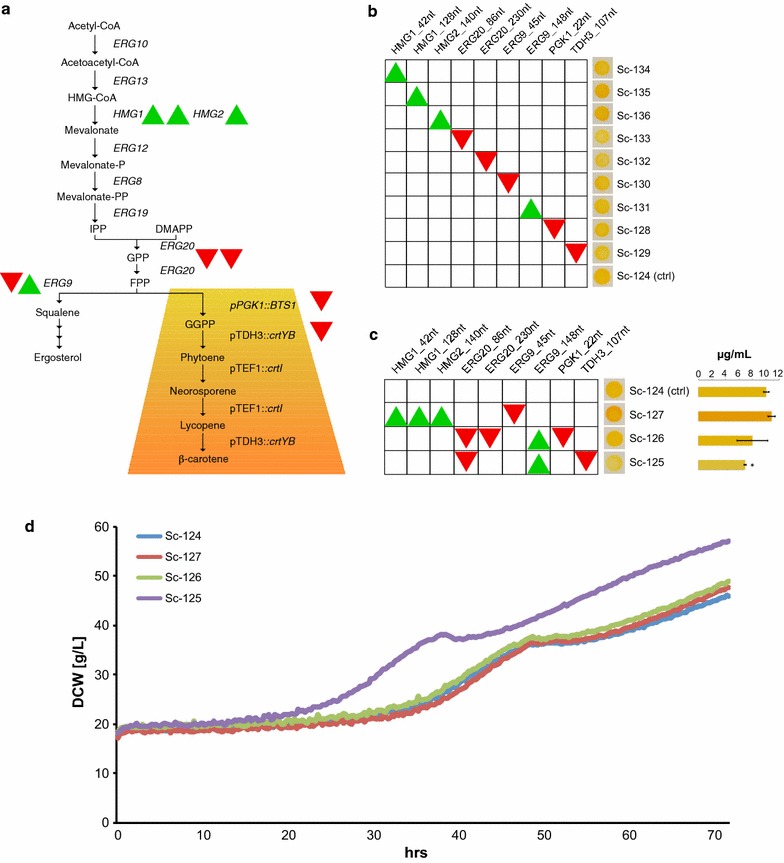
Transcriptional regulation changes carotenoid production. **a** Yeast MVA pathway coupled to carotenoid production. *Red triangles* (repression with PCP-Mxi1) and *green triangles* (activation with MCP-VPR) illustrate the orientation of transcriptional regulation on selected promoters. pHMG1 and pERG20 were targeted for regulation in up to two sites simultaneously, and pERG9 was either activated or repressed in different modules. **b** Nine strains (Sc-128 to Sc-136) are shown with single scRNAs targeting indicated promoters for regulation. *Triangles* indicate repression or activation as in **a**. Representative phenotypes including control (Sc-124) are presented next to respective constructs. **c** *Left* Individual scRNAs presented in **b** were multiplexed into three different designs. *Triangles* indicate reprogramming as in **a**. *Right* Quantification of β-carotene titers for each strain is presented. Values are shown as mean ± S.D. from three (n = 3) biological replicate experiments (*p < 0.01). **d** Growth assay depicting dry cell weight (DCW) per liter (g/L) as function of time for strains shown in **c** expressing multiplex scRNA designs for regulating carotenoid production. *Blue* Sc-124. *Purple* Sc-125. *Green* Sc-126. *Red* Sc-127. Presented data are averages of three biological replicates

In order to translate the observed effects from our reporter assays into reprogramming biosynthetic pathway flux, we next decided to investigate if the best-performing gRNAs would enable regulation of flux towards biosynthesis of either carotenoid or TAGs. With respect to the former, we first coupled the MVA pathway to carotenoid production using the strong glycolytic promoters, pPGK1 and pTDH3 already tested (Fig. 2a), to drive the expression of the gene encoding geranylgeranyl diphosphate synthase (*BTS1*) and those encoding *Xanthophyllomyces dendrorhous* lycopene cyclase/phytoene synthase (*crtYB* and *crtI*), respectively (Fig. 4a). The heterologous carotenoid pathway competes with sterol synthesis for the common precursor FPP, and repression of pERG9 has previously been shown to direct pathway flux towards isoprenoid production, while tuning the activity of pERG20 and pHMG1 has also been used to perturb MVA pathway flux [7, 29].

Next we analysed the effect of single gRNA expression on carotenoid production as inferred by phenotypic changes, using our 9 best-performing scRNAs targeting pERG20, pTDH3, pERG12, pERG9, pPGK1, and pHMG1 (Fig. 4b). From our experiment, the two scRNAs targeting dCas9 and PCP-Mxi1 to pERG20 at position TSS-86 and pTDH3 at position TSS-107, were able to modestly decrease the carotenoid-associated orange phenotype (Fig. 4b). *Vice versa*, targeting dCas9 and MCP-VPR to pHMG2 at position TSS-140 enabled modest increase in the carotenoid-associated phenotype (Fig. 4b). To further boost transcriptional impact for re-directing pathway flux, we multiplexed a subset of single scRNAs (Fig. 4c). We based our constructs on our results obtained from single scRNAs, and previous reports showing transcriptional impact on production as a result of transcriptionally regulating pERG20, pERG9, and pHMG1 [7–9]. Additionally we targeted the repression of either pPGK1 or pTDH3 (Figs. 2a, 4c). When multiple scRNAs were introduced, we observed phenotypic changes relative to our control strain (Sc-124) for two of three constructs. In strain Sc-127, with scRNAs activating pHMG1 and pHMG2 while simultaneously repressing pERG9, the intensity of the carotenoid pigmentation increased in correlation with the design. For strain Sc-126, carrying scRNAs for repression of pERG20 and pPGK1, while simultaneously activating pERG9, no change of pigmentation was observed. However, for both strains Sc-126 and Sc-127, no significant changes were observed in beta-carotenes (*p* = 0.12 and *p* = 0.05, respectively) (Fig. 4c). Contrastingly, for strain Sc-125, carrying scRNAs for repression of pERG20 and pTDH3 and simultaneous activation of pERG9, a lighter yellow phenotype was observed which correlate with significant lowered beta-carotene content (*p* = 8.03E−06) (Fig. 4c). To rule out the possibility that regulated production was merely an attribute from reduced growth rate, we investigated the growth rate for all four strains (Fig. 4d). Here, we observed no negative effect on growth rates in any of the tested strains. The OD increase over time was similar between most strains including the control strain, except for Sc-125 that exhibited improved growth.

**Fig. 5:**
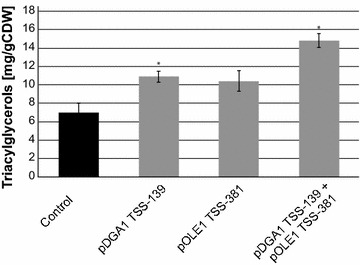
Triacylglycerol quantification after induced transcriptional regulation. TAG production was engineered in three strains; single gRNAs targeting positions TSS-139 on pDGA1 (pERA-122 in Sc-164) and TSS-381 on pOLE1 (pERA-112 in Sc-162) and multiplexed expression of both gRNAs coupled to dCas9-VPR (pERA-132 in Sc-166). The strains were cultivated in shake flasks for 24 h at 200 rpm, 30 °C. Results are shown as mean ± S.D. from three (n = 3) biological replicate experiments with asterisks indicating significant changes in triacylglycerol content relative to the control strain (*p < 0.05)

In our second test-bed, we investigated TAG biosynthesis by regulating pOLE1 and pDGA1. To increase the precursor supply towards fatty acids and TAGs, a constitutively active version acetyl coenzyme A carboxylase (ACC1) was expressed as previously reported [37]. When single best-performing gRNAs for pOLE1 and pDGA1 were expressed together with dCas9-VPR, TAG levels increased 1.5-fold over WT, while double expression of gRNAs lead to >twofold increase after twenty-four hours (Fig. 5). While we were able to increase TAGs production, reduction was not possible with the current setup (Additional file 6: Figure S5).

Taken together, the results from both the test beds demonstrate that transcriptional reprogramming of both linear and branched metabolic pathways is possible using the two different dCas9-mediated strategies.

## Discussion

Native regulatory transcriptional networks are balanced to maintain cellular homeostasis and adapt towards defined environments [38, 39]. For the purpose of cell factory engineering this offers robustness, yet can impose challenges when introducing non-native biosynthetic pathways requiring high flux from native metabolic routes [29]. Deletion of native genes encoding metabolic pathway steps that compete with the heterologous metabolic pathway is one approach used to perturb metabolic fluxes towards the product(s) of interest. Yet, this can be challenging both in terms of numbers of genes to knock-out, but also in terms of maintaining cell growth if targeting essential genes. In addition to gene knock-out strategies, re-directing metabolic fluxes by tuning the expression of essential genes encoding competing pathway steps is another strategy often used to improve biobased production. This has traditionally been accomplished by integrating inducible or repressible promoters to control the expression of flux controlling genes amenable to transcriptional regulation [7, 29, 40]. More recently, CRISPR/dCas9 has been adopted for redirecting metabolic fluxes through transcriptional regulation of single and multiple genes [21, 22, 24, 41]. With the results presented in our study using inducible CRISPR/dCas9 and the scRNA-mediated combinatorial reprogramming for scaffolding synthetic transcription machineries, metabolic engineers now have more tools to allow for inducible and multiplex control of expression of essential target genes, which simultaneously can sustain growth. In our study we observed no negative effects on growth when activating the inducible or constitutive CRISPR/dCas9 systems (Additional file 2: Figure S1; Fig. 4d). This highlights the future potential of dCas9 as a simple tool for balancing between production of interest and growth, as also reported in *E. coli* [41]. Also, though *S. cerevisiae* is neither a natural producer of carotenoids, nor a preferred microbial cell factory for TAG biosynthesis, the renowned orthogonality of CRISPR/dCas9 should be amenable for implementation in natural carotenoid and high-TAG producing hosts like *Xanthophyllomyces dendrorhous* and *Yarrowia lipolytica*, respectively. Indeed, CRISPR/Cas9 has alsready been demonstrated in *Yarrowia lipolytica* [42].

In our study we have successfully tested two systems for transcriptional reprogramming by the use of well-characterised phenotypes and selection, allowing us to retrieve variants harboring the desired properties (Figs. 4b, c, 5). Still several features need to be improved in order to predictably engineer transcriptional reprogramming for desired phenotypes using RNA-guided dCas9. Firstly, in our study we have used currently available software for designing gRNAs focusing on (1) minimal off-target effects, (2) proximity of TSS, and (3) positioning within regions of low nucleosomal positioning. However, from our data it is evident that even state-of-the-art software tools are not able to reliably predict how specific gRNAs and scRNAs quantitatively interfere with the activity of a native promoter. For instance, though several studies have reported correlation between the relative expression level and the proximity of the gRNA position to TSS [23, 30], this correlation is not evident when assessing our full library of tested scRNAs primarily targeting dcas9 to the TSS-200 to TSS+1 window (Fig. 2b). Also, though we observed a strong correlation between the degree of nucleosomal occupancy of the scRNA target site and the transcriptional impact for pHMG1, for most of our 14 target promoters this correlation was not prominent (Additional file 5: Figure S4). However, with respect to nucleosome positions, it is worth mentioning that information obtained from S288C as reported in Kaplan et al. [33], may not be shared with the CEN.PK genome. In our study, the lack of data on nucleosome positioning in CEN.PK could have hampered identification of appropriate target sites for scRNAs. Another feature which should be considered for optimizing transcriptional reprogramming is using other dCas9 variants. Recently Cas9/dCas9 hotspots for engineering were mapped [43], and these present an opportunity to build additional domains into dCas9. If transcriptional regulation is a matter of titration of transcriptional effectors, introducing such domains into dCas9 hotspots could potentially facilitate, and further potentiate transcriptional regulation [43]. Such a modified dCas9 could function in addition to direct fusions between dCas9 and Mxi1, and in particular VPR, to enhance transcriptional regulation. Similarly, the use of smaller Class 2 RNA-guided nuclease effectors may improve the transcriptional activation potential by limiting potential sterical hindrance of the RNA polymerase [44]. Having said this, once identified, effective gRNAs enable transcriptional reprogramming over time as evidenced from our time-resolved reporter assays (Fig. 3c; Additional file 3: Figure S2).

Finally, though both strategies tested in this study performed similarly on pHMG1, it is evident that the systems offer different advantages, which should be considered depending on the application. The inducible system can easily be turned into a one-plasmid strategy, which maintains inducible control over one or more gRNA expression cassettes. Likewise, this system offers external tuning of expression. The constitutive system on the other hand, can also be greatly expanded as recently demonstrated by the coupling of the PP7 RNA scaffold to bacterial small-molecule-regulated protein degron domains in order to support conditional activation [45]. This additional layer to scaffold-mediated transcriptional regulation makes it a very powerful tool for future use.

Taken together, this work has provided valuable information on transcriptional regulation using dCas9 in direct and indirect fusions to transcriptional effectors in yeast. Though there is a need for further refining the tools, our results provide the framework for future work on pathway regulation in yeast and further investigations for improving computer-aided design rules for gRNA design.

## Conclusion

In conclusion our study shows a similar performance of two different dCas9-mediated strategies for control of gene expression. Following testing >100 gRNAs we used combinations of the best-performing ones to reprogram yeast cells towards changes in production of carotenoids and TAGs. As some of the gRNA targets used in this study are essential genes, the design and selection of efficient gRNAs identified in this study are promising valves for prospecting, balancing and optimization of pathway flux without the need for genome engineering. Moreover, the testbeds used in the study are of strong interest for biobased production of value-added chemicals and fuels, and hence should be of of broad interest to the metabolic engineering community.

## Methods

### Strain and plasmid construction

Plasmids, strains and primers used in this study are listed in Additional file 7: Table S1, Additional file 8: Table S2, Additional file 9: S3 1. Oligonucleotides and gBlocks were ordered from IDT and Eurofins. All fragments obtained by PCR were gel- or column purified (Nucleospin^®^ Gel and PCR Clean-up columns) before cloning, and resulting plasmids were verified by sequencing (Eurofins). Yeast transformations were done using lithium acetate and PEG3350, and genomic integrations were performed with various helper plasmids and pre-expressed iCas9 from plasmid pCT (Addgene #60620) and plated on Sc-Leu+cloNAT. Strains were cured for pCT and helper plasmids after genome engineering and before proceeding to transcriptional regulation using dCas9.

EasyClone-MarkerFree vectors pCfB2909, pCfB3035, pCfB3037 and helper plasmids pCfB3042, pCfB3046, and pCfB3050 as well as genomic integration verification primers were adapted as previously described [46]. Yeast strains were plated according to auxotrophies and plasmid markers. *Mxi1* [18] and *PCP* [24] were fused with pADH1 into pCfB3035 to create a transcription-repressing construct for genomic integration. pADH1 was obtained by PCR amplification of genomic DNA with primers pADH1_fw and pADH1_rv. Mxi1 was PCR amplified from Addgene plasmid #46921 with primers EDJ-126 and EDJ-127. The PP7 RNA scaffold-binding protein PCP was PCR amplified from gBlock gEDJ-9 with primers EDJ-124 and EDJ-125. *Mxi1* was USER assembled with pADH1 and *PCP* into *Sfa*AI/Nb.*Bsm*I pre-digested vector pCfB3035, resulting in plasmid pEDJ-22. The PCP-Mxi1 fusion was intervened by a 3× GS linker. The transcriptional enhancer [25] was obtained from Addgene plasmid #63801 by PCR with primers EDJ-098 and EDJ-099. The 2× (wt + f6) RNA scaffold-binding protein MCP [24] was obtained by PCR amplification of gEDJ-7 using primers EDJ-096 and EDJ-097. *VPR* was USER assembled with pADH1 and *MCP* into *Sfa*AI/Nb.*Bsm*I pre-digested vector pCfB3037, resulting in plasmid pEDJ-87. In this construct, a 1× GS linker and SV40-NLS separate MCP from VPR. pEDJ-22 and pEDJ-87 were digested by *Not*I and purified on column before transformation. Digests were sequentially integrated using helper plasmids pCfB3042 and pCfB3046, respectively, into *S. cerevisiae* strain CEN.PK102-3A. The resulting integrations into EasyClone-MarkerFree sites X-4 (pEDJ-22) and XI-5 (pEDJ-87) defined a new strain, Sc-10. Integration at EasyClone-MarkerFree site X-4 was verified by genomic integration verification primer pairs 2221 and 905, 2220 and 906, 905 and 906, and integration at EasyClone-MarkerFree site XI-5 was verified by genomic integration verification primers 2221 and 8418, 2220 and 8419, and 8418 and 8419.

Next, promoter-*GFP* fusions were made with 12 individual yeast promoters obtained by using primers ERA-1 to ERA-24 on *S. cerevisiae* strain CEN.PK113-7D genomic DNA, and *GFP* obtained by PCR with primers GFPopt_fw and GFPopt_rv on p416TEF-GFP. Promoter-*GFP* fusions were USER assembled into *Sfa*AI/Nb.*Bsm*I pre-digested vector pCfB2909. Resulting plasmids pERA-1 to pERA-12 were digested by *Not*I and integrated in yeast strain Sc-10 into EasyClone-MarkerFree site XII-5 using helper plasmid pCfB3050, thus constituting strains Sc-11 to Sc-22. Plasmids pERA-13 and pERA-14 were assembled by USER cloning and express *dCas9* from pTDH3 and pTEF1, respectively. The *dCas9* ORF was amplified by PCR from plasmid Addgene (#63801) with primers EDJ-204 and EDJ-205. pTDH3 was generated by PCR amplification of genomic DNA with primers Tdh3p_fw and Tdh3p_rv, and pTEF1 was obtained from amplification of p0029 [47] with primers Tef1p_fw and Tef1p_rv. Assemblies were done into plasmid pRS415U pre-digested with *Sfa*AI/Nb.*Bsm*I to constitute the final plasmids pERA-13 and pERA-14. Plasmid pERA-13 was transformed into Sc-11 to Sc-14 and Sc-16 to Sc-22 to yield the new strains Sc-23 to Sc-26 and Sc-28 to Sc-34. Sc-15 was transformed with pERA-14, making up strain Sc-27. Addgene plasmids pJZC603 (#62317) [24] carrying the 2xPP7 scRNA scaffold and pJZC588 (#62315) [24] containing the 2× (wt + f6) scRNA scaffold were inversely PCR amplified with forward primer ERA-37 and reverse primer TJOS-20 [48] and blunt-end ligated by T4 DNA ligase (NEB) and digested by *Dpn*I before transformation into *E. coli* strain DH5-α and plasmid purification, resulting in plasmids pEDJ-51 and pEDJ-52, respectively. Primers EDJ-216 and EDJ-221 amplified the scRNA cassettes from pEDJ-51 and pEDJ-52 that were subsequently used in a USER-assembly into *Sfa*AI/Nb.*Bsm*I pre-digested USER plasmid p0054 [47], generating plasmids pERA-39 and pERA-40, respectively. Plasmids pEDJ-51 and pEDJ-52 served as templates for forward primers ERA-25 to ERA-68 paired with reverse primer TJOS-20 to generate plasmids pERA-15 to pERA-102 harboring gRNA1 to gRNA44 attached to 2× PP7 or 2× (wt + f6) scaffolds. Plasmids pERA-15 to pERA-44 and pERA-53 to pERA-102 were co-transformed with pERA-13 into strains Sc-11 to Sc-14 and Sc-16 to Sc-22, respective to integrated promoter-*GFP* constructs, to create strains Sc-35 to Sc-64 and Sc-73 to Sc-122. pERA-45 to pERA-52 were co-transformed with pERA-14 into strain Sc-15 to create strains Sc-65 to Sc-72. Resulting transformants were used for fluorescence analysis. Multiplex scRNA plasmids for regulating carotenoid production were generated as described [48] and outlined below, using PhusionU for polymerization.

For generation of multiplex gRNA plasmid pERA-103, plasmid pERA-86 served as template for primers EDJ-216 and EDJ-217, plasmid pERA-67 as template for primers EDJ-218 and EDJ-219, and plasmid pERA-48 as template for primers EDJ-220 and EDJ-221. All three fragments were gel purified and USER-assembled into *Sfa*AI/Nb.*Bsm*I digested plasmid p0054. To generate the multiplex gRNA plasmid pERA-104, plasmid pERA-86 served as template for primers EDJ-216 and EDJ-217, plasmid pERA-85 as template for primers EDJ-218 and EDJ-226, plasmid pERA-29 as template for primers EDJ-219 and EDJ-225, and plasmid pERA-67 as template for primers EDJ-220 and EDJ-221. Assembly was performed as for generating pERA-103. For generation of multiplex gRNA plasmid pERA-105, plasmid pERA-95 served as template for primers EDJ-216 and EDJ-217, plasmid pERA-96 as template for primers EDJ-218 and EDJ-226, plasmid pERA-62 as template for primers EDJ-219 and EDJ-225, and plasmid pERA-100 as template for primers EDJ-220 and EDJ-221. Assembly was done as for generating plasmids pERA-103 and pERA-104. All gRNA sequences are listed in Additional file 1: Table S4.

Strain Sc-10 was further engineered to express the carotenoid pathway previously described [27]. The constructs were made as according to [46]. pPGK1 and *BTS1* was USER cloned into pCfB2903 and called pTAJAK-182. pTDH3 and *crtI* was USER cloned into pCfB3034 and called pTAJAK-183. pTEF1 and *crtYB* was USER cloned into pCfB3039 and called pTAJAK-184. After *Not*I digestion, all three constructs were transformed into Sc-10 pre-expressing pCT and with pCfB3051 for Cas9-guided integration. pERA-13 and p0054 co-transformed Sc-123 to yield Sc-124 serving as control (no regulation) on carotenoid production. pERA-14 and pERA-103 co-transformed Sc-123 to give Sc-125. pERA-13 transformed Sc-123 with pERA-104 or pERA-105 to give strains Sc-126 or Sc-127, respectively. pERA-14 cotransformed Sc-123 with pERA-48 resulting in Sc-129. Sc-123 was also transformed by pERA-13 in combination with pERA-29, pERA-62, pERA-67, pERA-85, pERA-86, pERA-95, pERA-96, or pERA-100 making up strains Sc-128 and Sc-130 to Sc-136.

The plasmid pERA-109 was constructed by amplifying the VPR using VPR-F and VPR-R primers from pAG414GPD-dCas9-VPR plasmid (Addgene #63801). The PCR fragment was treated with *Dpn*I, gel purified and inserted with Gibson assembly [49] into plasmid pRS414-Tef1-NLS-dCas9-Cyc1 [23] at the *Pac*I site. pOLE1 was amplified from CEN.PK113-11C genomic DNA using OLE1promoter-F and OLE1promoter-R pair of primers. GFP was amplified from p416TEF-GFP plasmid using OLE1gfp-F and GFP-R pair of primers. The vector p413-TEF (ATCC^®^ 87362™) was amplified with pair of primers annealing outside the TEF promoter region using p413-F and p413-R primers. pOLE1, GFP and p413 PCR fragments were cloned together using Gibson Assembly to create pERA-110. pDGA1 was amplified from CEN.PK113-11C genomic DNA. *GFP* was amplified from p416TEF-GFP using DGA1gfp-F and GFP-R. pDGA1, GFP, and p413-TEF PCR fragments were cloned together using Gibson Assembly to make pERA-111. gRNAextender-F/R gRNAextender-R primers amplified ERA-69 to ERA-79 that were subsequently cloned into pERA-109 and dCas9-Mxi according to Smith et al. [23], constituting pERA-112 to pERA-131 harboring gRNA45-gRNA54 and also pERA-134 and pERA-135 harboring gRNA39. Correct clones were confirmed with colony PCR using 3 primers (62-pRPR1-*Not*I-fwd, 49-pRPR1_fwd and 74-RPR1t-5′-Rev). The multiplexed pOLE1/pDGA1 gRNA expression plasmid was built by amplifying from the plasmids pERA-112 using Multiplex1 and Multiplex2 pair of primers, and, pERA-113 using Multiplex3 and Multiplex4 pair of primers. The two PCR fragments were cloned into pERA-109 and dCas9-Mxi in *Not*I by Gibson assembly to generate pERA-132 and pERA-133 both harboring gRNA45 and gRNA46. Plasmid pERA-110 and pERA-111 were transformed into CEN.PK-11C to yield the new strains Sc-137 and Sc-138 respectively. Sc-137 was transformed with pERA-109 and pERA-112 to pERA-121 making up strain Sc-139 to Sc-149. Sc-138 was transformed with pERA-109 alone and with pERA-122 to pERA-131 individually, making up strains Sc-150 to Sc-160. For TAG quantification, CEN.PK-11C with HXT7p-ACC1**-CYC1t at X-2 site (Sc-180) was transformed with pERA-109, pERA-112, pERA-117, pERA-122, pERA-127, pERA-132 and pERA-133 making up strains Sc-161 to Sc-167. Primers OLE1-GFP_F/OLE1-GFP_R amplified template pERA-110 to obtain pOLE1-*GFP* that was transferred to pCfB2909. Co-transforming the resulting *Not*I prepared fragment with helper plasmid pCfB3050 integrated pOLE1-*GFP* in CEN.PK102-3A produced strain Sc-168. Plasmids pERA-109, pERA-112 and pERA-117 were transformed into Sc-168 making up strains Sc-169 to Sc-171. Strain Sc-172 was generated from integrating pHMG1-*GFP* exactly as for creating strain Sc-21, but using CEN.PK102-3A as host strain instead of Sc-10. Plasmids pERA-109, pERA-134 and pERA-135 were transformed into Sc-172 making up strains Sc-173 to Sc-175. Plasmids pERA-109, pERA-122 and pERA-127 were transformed into Sc-176 making up strains Sc-177 to Sc-179. Primers OLE1-GFP_F/OLE1-GFP_R amplified template pERA-110 to obtain pOLE1-*GFP*. The fragments were USER-assembled into plasmid pCfB2909 followed by *Not*I digestion and integration into strain Sc-10 at EasyClone-MarkerFree site XII-5 using helper plasmid pCfB3050, resulting in strain Sc-176. Plasmids pERA-107 and pERA-108 were made by inverse amplification of pERA-94 with primers TJOS-20 and gRNA45_F. Sc-176 was transformed by pERA-13 alone and with either pERA-107 or pERA-108 making up strains Sc-177, Sc-178 and Sc-179 that were used for benchmarking the constitutive and inducible systems.

### Activating the inducible system

For cultivations in shake flasks, 5 mL of minimal medium was inoculated with a single colony from selective medium and incubated at 200 rpm and 30 °C O/N. Subsequently, the pre-culture was used to inoculate 10 mL of minimal medium with 250 ng/mL of aTc in shake flasks without baffles and a total volume of 100 mL at an OD_600_ of 0.05.

### Flow cytometer analysis

Transformants carrying the dCas9-expression plasmids pERA-13 or pERA-14 only were inoculated in liquid Sc-Leu, and strains additionally carrying pERA-15 to pERA-102 were inoculated in liquid Sc-Leu-Ura and incubated at 30 °C with agitation O/N. Cultures were passed the next day, 24 h before analysis, in 10× dilutions to fresh media and incubated at 30 °C with shaking. Then 30 μL of each culture was transferred into 150 μL of Phosphate Buffer Saline (PBS) from Life Technologies for analysis. The Guava easyCyte™ from EMD Millipore was used to analyse GFP intensity from 10,000 single-cells per culture with a blue laser at 488 nm, and FlowJo software (TreeStar Inc.) was used for processing events. At least three biological replicates were analysed, and arithmetic mean fluorescense for each population was determined. Error bars correspond to the deviation between replicate cultures, and fold-changes were calculated by dividing means from ’activated’ cultures with ’control’ cultures.

### BioLector cultivations

DCW, OD and GFP signals were recorded using a BioLector (m2p-labs, Baesweiler, Germany). For this purpose, at least three biological replicates were picked from selective medium and each used to inoculate 3 mL of Synthetic Medium followed by O/N incubation at 30 °C and 200 rpm. Cultures were each used to inoculate 1 mL of SC at an OD_600_ of ~0.2 for the inducible system. Subsequently, the cultures were transferred into a 48-well microtiter plate (MTP-48-B FlowerPlate, m2p-labs) and incubated at 30 °C and 1000 rpm using the BioLector. The optical density was measured on-line in 15 min intervals at filter gain 20 for the inducible system and filter gain 10 for the constitutive system. The fluorescence signal was measured at the filter gain 50 for the inducible system and filter gain 100 for the constitutive system.

### Transcriptional start site localization

Locations of TSSs were obtained from Miura et al. [50] and annotated via http://lp2.github.io/yeast-crispri/ [23].

### Carotenoid extraction procedure

Four replicates from whole-plate scrapings were dissolved in 5 mL Sc-Leu-Ura and incubated with shaking (250 rpm) at 30 °C for 72 h. Outgrown cultures were centrifuged at 3000 rpm for 3 min and supernatant discarded by pouring. Remaining supernatant was used to resuspend cell pellet and was transferred to 1.5 mL Eppendorf tubes. The tubes were centrifuged at 3000 rpm for 3 min and supernatant removed by pipetting. Cell pellet was resuspended in 250 μL Glucanex (Sigma-Aldrich) to the final concentration 1 mg/25 μL and incubated at 37 °C for 30 min. 300 μL hexane (Sigma-Aldrich) was added to each tube, and total volume was transferred to a 2 mL screw cap micro tube (Sarstedt) containing 0.25 mL acid washed glass beads (Sigma-Aldrich). Samples were run on a Precellys 24 homogenizer (Bertin Instruments) at 4 × 40 s at 6500 rpm. After extraction, hexane was separated from beads and pale cell pellet by centrifugation at 13,000*g* for 3 min. Hexane phase was transferred to a fresh 1.5 mL Eppendorf tube. 40 μL of each sample was transferred to a fresh 1.5 mL Eppendorf tube, and hexane was evaporated in a fume hood at room temperature. Dried content was resuspended in 1 mL 100% ethanol, and 150 μL was moved to a new vial for analysis.

### β-Carotene quantification

LC–MS analysis was performed with Orbitrap Fusion equipped with a Dionex Ultimate 3000 UHPLC pumping system (ThermoFisher Scientific, Waltham, MA, USA). Samples were held in the autosampler under 10.0 °C during the analysis. 7 μL of each sample was injected to a Supelco Discovery HS F5-3 HPLC column (2.1 × 150 mm, 3 μm), at a flow rate of 0.7 mL/min, 30.0 °C. Mobile phases A and B were 0.1% formic acid in water and acetonitrile, respectively. Elution was done with a 25 min multistep system. After 25% B for 3 min, a linear gradient started from 25% B to 100% B in 12 min, which was held for another 5 min and followed by re-equilibration to 25% B until 25 min. The column eluent flowed directly into the heated ESI probe of the MS which was held at 325 °C and a voltage of 3500 V. Profile data was collected in positive ion mode with resolution setting of 30 K and scan range (m/z) = 200–1000. Other MS setting parameters were as follows: sheath gas flow rate of 60 units, Aux gas flow rate of 20 units, sweep gas flow rate of 5 units, ion transfer tube temp was 380 °C, maximum injection time of 100 ms, S-lens RF level = 60 V, using 1 Microscans and AGC target = 200,000 counts. Carotene (m/z 536.4372, [M+H]^+^) was eluted at retention time 14.8 min. To quantify with bracketing calibration method, carotene standards with calibration ranging 1.0–30.0 µg/mL were prepared and measured together with all samples. Further processing was carried out using Thermo Xcalibur 3.0.

### TAGs quantification

Samples for lipid analysis were taken after 24 h of shake flask cultivation. Subsequently, the samples were centrifuged at 2000 rcf and the supernatant was discarded. The pellets were kept at −20 °C for 15 min and then freeze-dried using a Christ alpha 2–4 LSC (Christ Gefriertrocknungsanlagen, Osterode, Germany). For all samples 10 mg of freeze-dried biomass were transferred into the extraction tubes. 50 μg of Cholesterol (1 mg/mL): Internal standard was added in the samples and the blanks. 7 mL of CHCl3:MeOH (2:1) and N_2_ gas was flushed into the tube to remove the air. After vortexing the tubes for 1 min, the tubes were put into the microwave vessel containing 30 mL water inside and then placed in the microwave. Samples were heated from room temperature to 60 °C (within 6 min) and kept at 60 °C for 10 min. 1.7 mL of 0.73% NaCl were added after the samples were cooled down to room temperature. After vortexing for 20 s, the tubes were centrifuged at 3000 rpm for 5 min. The organic phase was transferred into clean extraction tubes. The sample were dried with MiVac Evaporator and re-suspended with 200 μL of CHCl_3_:MeOH (2:1). Samples were then analysed via HPLC-CAD as described by [51].

## Additional files

**Additional file 1: Table S4.** gRNA sequences and target information.

**Additional file 2: Figure S1.** Growth kinetics during transcriptional regulation of pHMG1 and pOLE1. Indicated strains were the same as in Fig. 1C targeted for regulation at pHMG1 or pOLE1, with controls (ctrl) expressing dCas9 and no gRNA. Growth was monitored over ~47 hours with a BioLector, and presented data is the average of three biological replicates. **A**. Strains that harbored the constitutive system were monitored for increasing dry cell weight (DCW) per liter (g/L) for controls (blue), activation with MCP-VPR (green), and repression with PCP-Mxi1 (red). **B**. Strains that contained the inducible system were cultured with 250 ng/mL aTc, and OD_600_ was monitored. Blue indicates control cultures (dCas9), green activation (dCas9-VPR), and red repression (dCas9-Mxi1).

**Additional file 3: Figure S2.** Time-dependent regulation of reporter gene expression. Data were obtained with a BioLector from the same cultures as used in Supplementary Fig. S1. Indicated strains were targeted at pHMG1 or pOLE1 as in Fig. 1C with controls (ctrl) expressing dCas9 and no gRNA. Data were collected for ~47 hrs and are presented as the average of three biological replicates. **A**. MFI from strains targeted with the constitutive system is presented per DCW/L (dry cell weight per liter) as a function of time. Blue; control. Green; activation (MCP-VPR). Red; repression (PCP-Mxi1). **B**. MFI from cultures added 250 ng/mL aTc to activate the inducible system is presented per OD (OD_600_) over time. Blue; control. Green; activation (dCas9-VPR). Red; repression (dCas9-Mxi1).

**Additional file 4: Figure S3.** CRISPR/dCas9-mediated regulation of twelve native yeast promoters. Twelve yeast promoters as indicated were targeted with scRNAs in a total of 88 strains for regulation at various positions. Deviations from ’no gRNA’ control for each promoter is shown on second axis (relative MFI), and promoters with indicated distances from scRNA hybridization sites to TSS+1 are listed on first axis. Dark grey bars represent regulation with MCP-VPR (activation) and light grey bars regulation with PCP-Mxi1 (repression). GFP emission was measured following 24 hrs incubation and MFI calculated. MFI values are shown as mean ± s.d. from three (n = 3) biological replicate experiments (* = *p* < 0.01).

**Additional file 5: Figure S4.** Transcriptional regulation vs. nucleosome occupancy. Relative MFI (deviation from ’no gRNA’ control) is shown on second axis vs. predicted nucleosome occupancy on first axis for 12 yeast promoters. Predicted nucleosome occupancies were based on Kaplan et al. [33] and are the averaged values between the 5’- and 3’-most occupancy values of gRNAs. Low predicted nucleosome occupancy scores relate to lower nucleosome densities at a given position. Dark grey symbolizes regulation with MCP-VPR (activation) and light grey with PCP-Mxi1 (repression). Results are based on at least three biological replicates.

**Additional file 6: Figure S5.** Triacylglycerol quantification after induced transcriptional regulation for both activator and repressor. TAG production was engineered in three yeast strains; single gRNAs targeting position TSS-139 on pDGA1 (Sc-164 and Sc-165; pERA-122, pERA-127) and TSS-381 on pOLE1 (Sc-162 and Sc-163: pERA-112, pERA-117) and multiplexed expression of both gRNAs coupled to dCas9-VPR (Sc-166 and Sc-167: pERA-132, pERA-133). The strains were cultivated in shake flasks for twenty-four hours at 200 rpm, 30 °C. Results are based on three biological replicates. Significant changes are indicated by asterisk.

**Additional file 7: Table S1.** Yeast strains.

**Additional file 8: Table S2.** Plasmids.

**Additional file 9: Table S3.** Primers.
