# Supplementary figures and images for: Transcriptional reprogramming in yeast using dCas9 and combinatorial gRNA strategies
Source: Microb Cell Fact. 2017 Mar 15;16:46. doi: 10.1186/s12934-017-0664-2 (PMC5353793; doi:10.1186/s12934-017-0664-2)

A

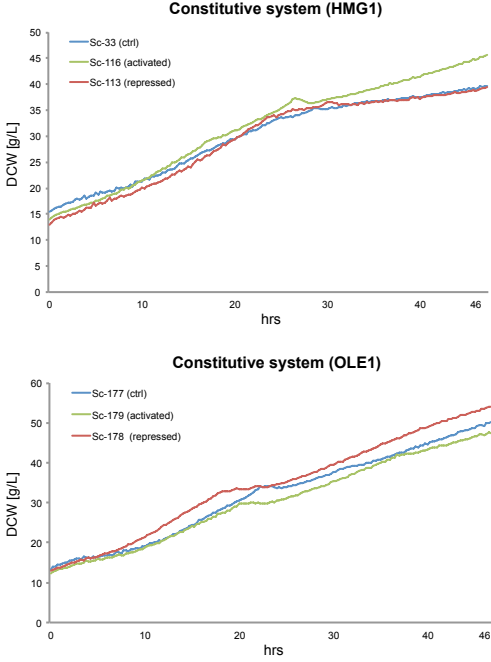

B

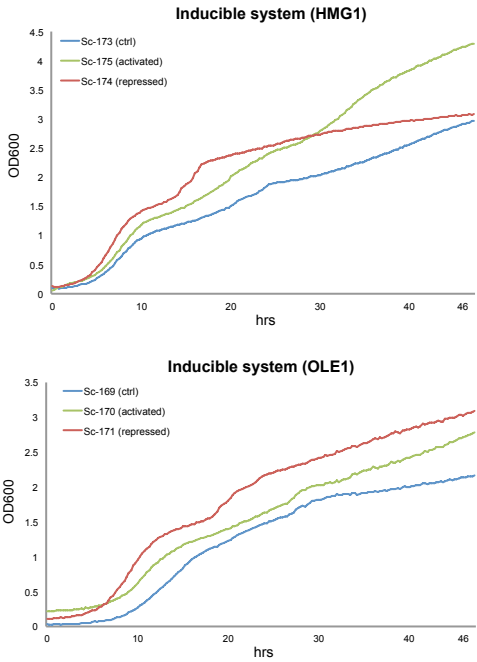

Supplementary Figure S1.

Supplement: Supplementary file 2 — Additional file 2: Figure S1. Growth kinetics during transcriptional regulation of pHMG1 and pOLE1. Indicated strains were the same as in Fig. 1C targeted for regulation at pHMG1 or pOLE1, with controls (ctrl) expressing dCas9 and no gRNA. Growth was monitored over ~47 hours with a BioLector, and presented data is the average of three biological replicates. A. Strains that harbored the constitutive system were monitored for increasing dry cell weight (DCW) per liter (g/L) for controls (blue), activation with MCP-VPR (green), and repression with PCP-Mxi1 (red). B. Strains that contained the inducible system were cultured with 250 ng/mL aTc, and OD600 was monitored. Blue indicates control cultures (dCas9), green activation (dCas9-VPR), and red repression (dCas9-Mxi1). [file 12934_2017_664_MOESM2_ESM.pdf]

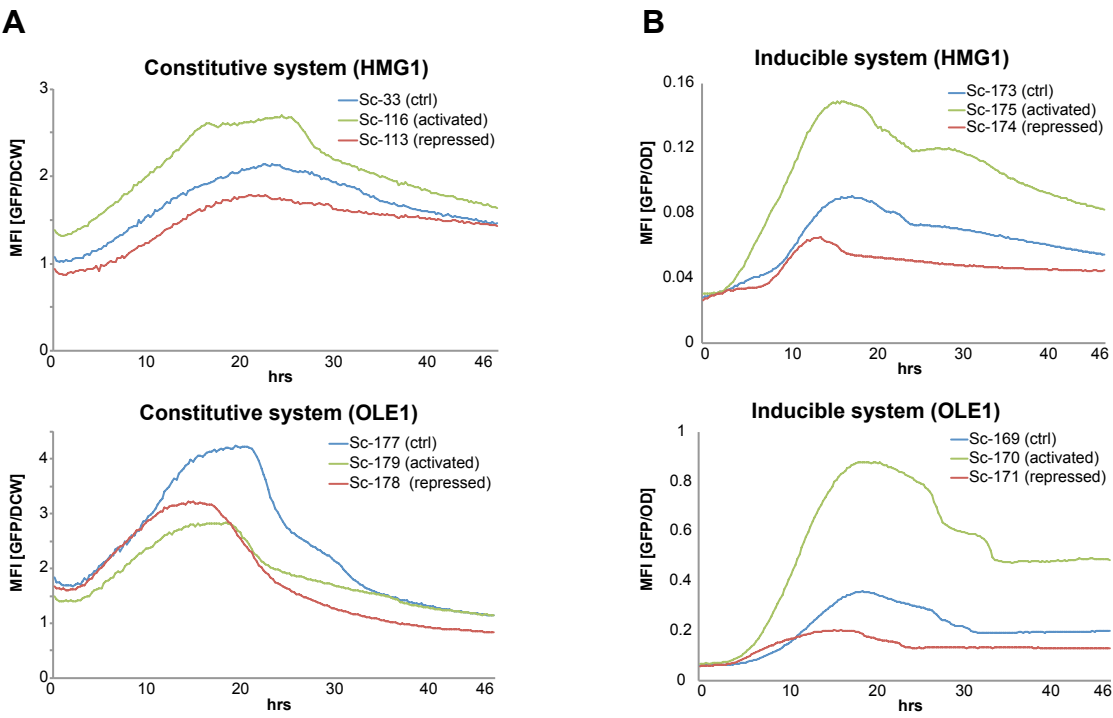

Supplementary Figure S2.

Supplement: Supplementary file 3 — Additional file 3: Figure S2. Time-dependent regulation of reporter gene expression. Data were obtained with a BioLector from the same cultures as used in Supplementary Fig. S1. Indicated strains were targeted at pHMG1 or pOLE1 as in Fig. 1C with controls (ctrl) expressing dCas9 and no gRNA. Data were collected for ~47 hrs and are presented as the average of three biological replicates. A. MFI from strains targeted with the constitutive system is presented per DCW/L (dry cell weight per liter) as a function of time. Blue; control. Green; activation (MCP-VPR). Red; repression (PCP-Mxi1). B. MFI from cultures added 250 ng/mL aTc to activate the inducible system is presented per OD (OD600) over time. Blue; control. Green; activation (dCas9-VPR). Red; repression (dCas9-Mxi1). [file 12934_2017_664_MOESM3_ESM.pdf]

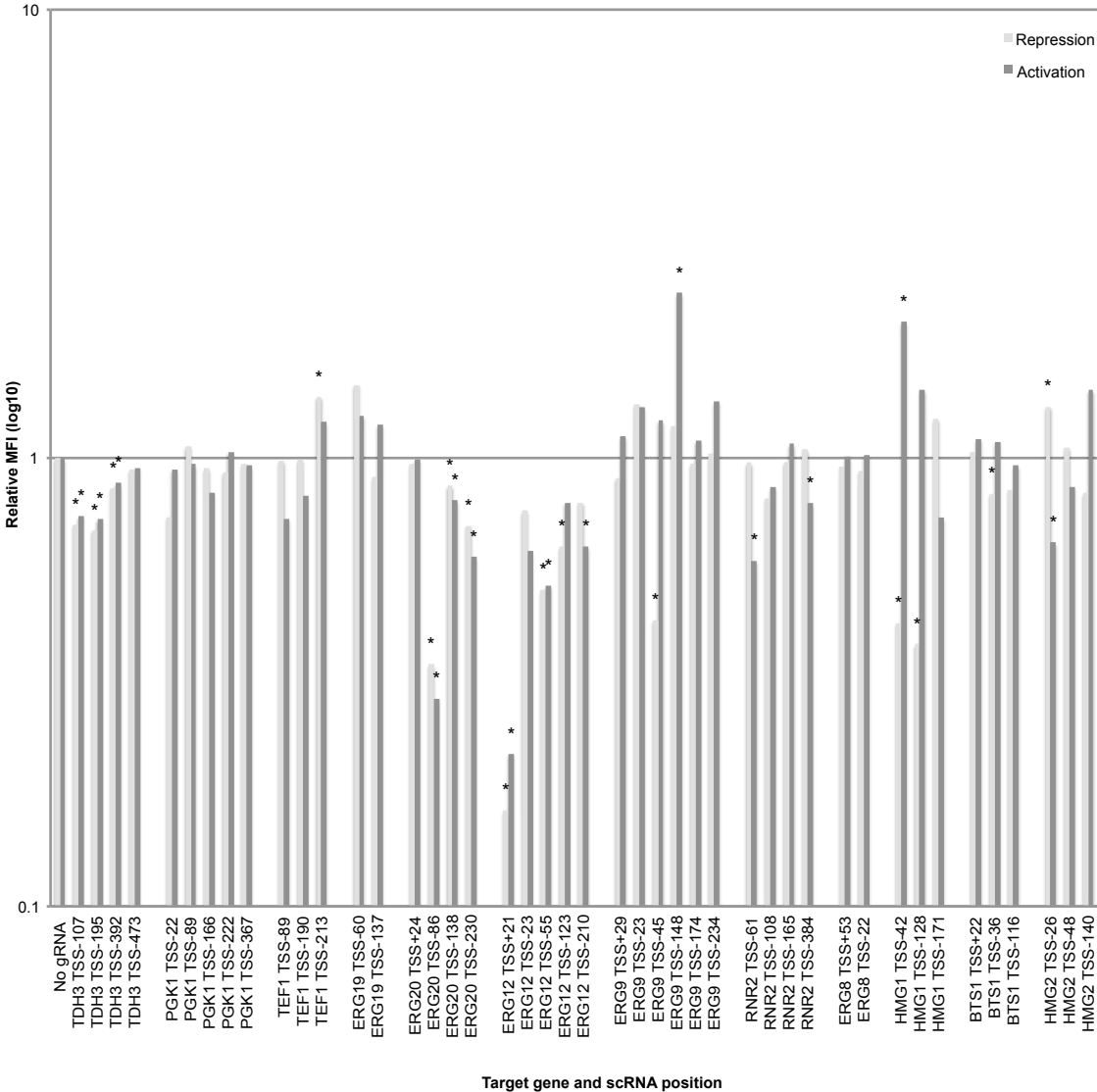

Supplementary Figure S3.

Supplement: Supplementary file 4 — Additional file 4: Figure S3. CRISPR/dCas9-mediated regulation of twelve native yeast promoters. Twelve yeast promoters as indicated were targeted with scRNAs in a total of 88 strains for regulation at various positions. Deviations from ’no gRNA’ control for each promoter is shown on second axis (relative MFI), and promoters with indicated distances from scRNA hybridization sites to TSS+1 are listed on first axis. Dark grey bars represent regulation with MCP-VPR (activation) and light grey bars regulation with PCP-Mxi1 (repression). GFP emission was measured following 24 hrs incubation and MFI calculated. MFI values are shown as mean ± s.d. from three (n = 3) biological replicate experiments (* = p < 0.01). [file 12934_2017_664_MOESM4_ESM.pdf]

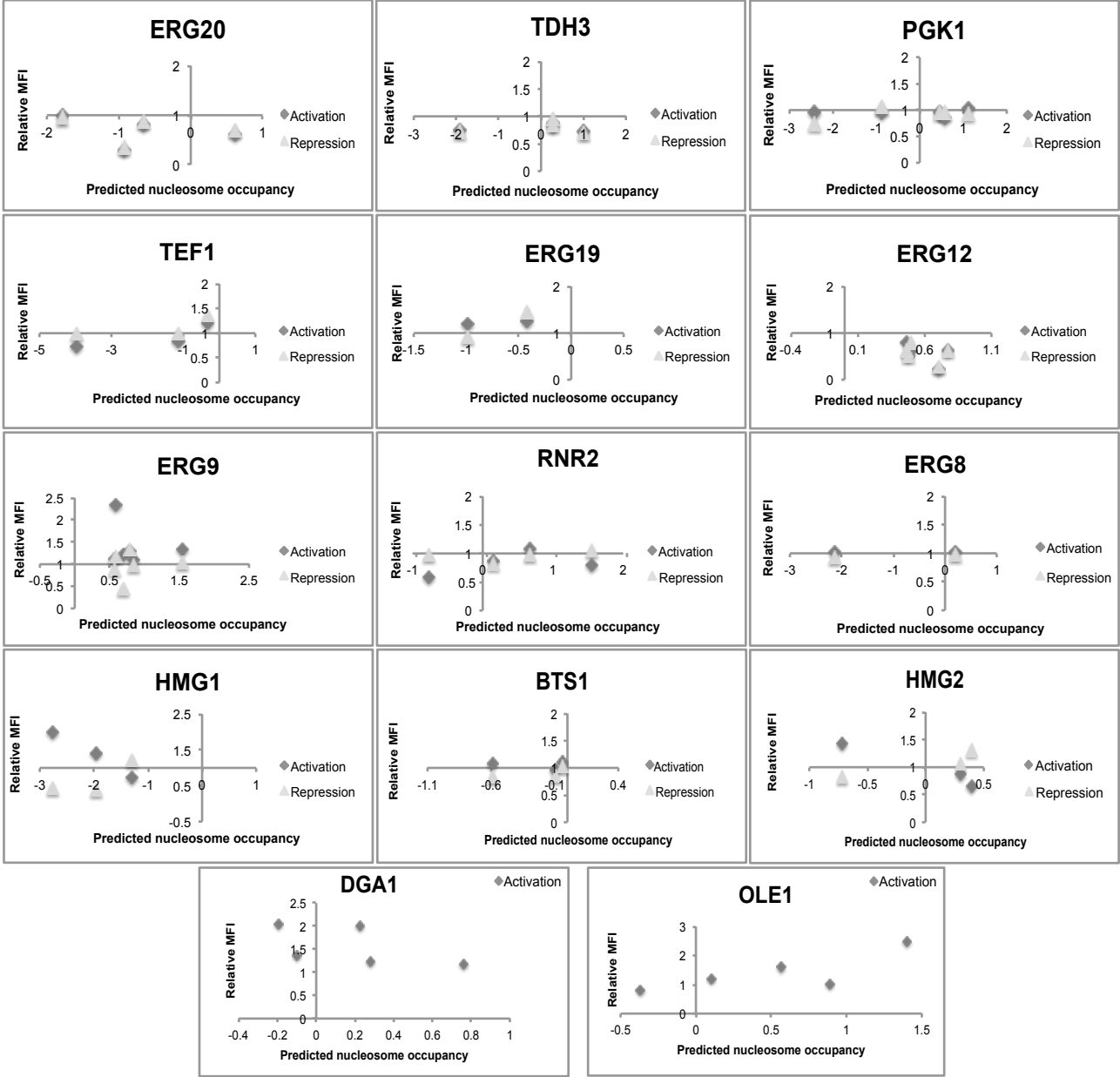

Supplementary Figure S4.

Supplement: Supplementary file 5 — Additional file 5: Figure S4. Transcriptional regulation vs. nucleosome occupancy. Relative MFI (deviation from ’no gRNA’ control) is shown on second axis vs. predicted nucleosome occupancy on first axis for 12 yeast promoters. Predicted nucleosome occupancies were based on Kaplan et al. [33] and are the averaged values between the 5’- and 3’-most occupancy values of gRNAs. Low predicted nucleosome occupancy scores relate to lower nucleosome densities at a given position. Dark grey symbolizes regulation with MCP-VPR (activation) and light grey with PCP-Mxi1 (repression). Results are based on at least three biological replicates. [file 12934_2017_664_MOESM5_ESM.pdf]

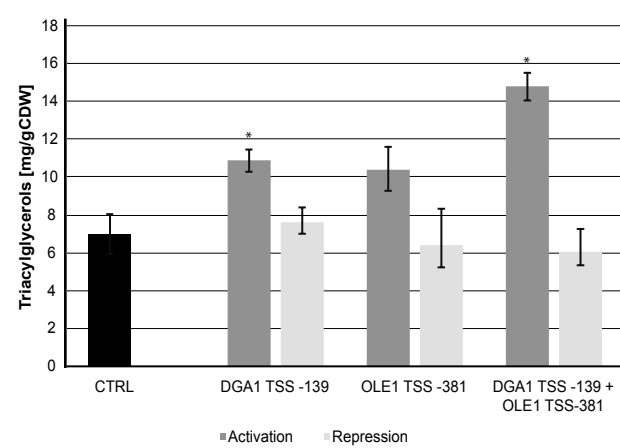

**Supplementary Fig. S5**

Supplement: Supplementary file 6 — Additional file 6: Figure S5. Triacylglycerol quantification after induced transcriptional regulation for both activator and repressor. TAG production was engineered in three yeast strains; single gRNAs targeting position TSS-139 on pDGA1 (Sc-164 and Sc-165; pERA-122, pERA-127) and TSS-381 on pOLE1 (Sc-162 and Sc-163: pERA-112, pERA-117) and multiplexed expression of both gRNAs coupled to dCas9-VPR (Sc-166 and Sc-167: pERA-132, pERA-133). The strains were cultivated in shake flasks for twenty-four hours at 200 rpm, 30 °C. Results are based on three biological replicates. Significant changes are indicated by asterisk. [file 12934_2017_664_MOESM6_ESM.pdf]
